# Superior semicircular canal dehiscence in East Asian women with osteoporosis

**DOI:** 10.1186/1472-6815-12-8

**Published:** 2012-07-25

**Authors:** Alexander Yu, Douglas L Teich, Gul Moonis, Eric T Wong

**Affiliations:** 1 South Cove Community Health Center, Boston, MA, USA; 2 Department of Neurology, Beth Israel Deaconess Medical Center, Boston, MA, USA; 3 Section of Neuroradiology/Department of Radiology, Beth Israel Deaconess Medical Center, Boston, MA, USA

**Keywords:** Vertigo, Osteoporosis, Superior semicircular canal, Canal dehiscence, Neurotology

## Abstract

**Background:**

Superior semicircular canal dehiscence (SSCD) may cause Tullio phenomenon (sound-induced vertigo) or Hennebert sign (valsalva-induced vertigo) due to the absence of bone overlying the SSC. We document a case series of elderly East Asian women with atypical SSCD symptoms, radiologically confirmed dehiscence and concurrent osteoporosis.

**Methods:**

A retrospective record review was performed on patients with dizziness, vertigo, and/or imbalance from a neurology clinic in a community health center serving the East Asian population in Boston. SSCD was confirmed by multi-detector, high-resolution CT of the temporal bone (with Pöschl and Stenvers reformations) and osteoporosis was documented by bone mineral density (BMD) scans.

**Results:**

Of the 496 patients seen in the neurology clinic of a community health center from 2008 to 2010, 76 (17.3%) had symptoms of dizziness, vertigo, and/or imbalance. Five (6.6%) had confirmed SSCD by multi-detector, high-resolution CT of the temporal bone with longitudinal areas of dehiscence along the long axis of SSC, ranging from 0.4 to 3.0 mm, as seen on the Pöschl view. Two of the 5 patients experienced motion-induced vertigo, two fell due to disequilibrium, and one had chronic dizziness. None had a history of head trauma, otologic surgery, or active intracerebral disease. On neurological examination, two patients had inducible vertigo on Dix-Hallpike maneuver and none experienced cerebellar deficit, Tullio phenomenon, or Hennebert sign. All had documented osteoporosis or osteopenia by BMD scans. Three of them had definite osteoporosis, with T-scores < −2.5 in the axial spine, while another had osteopenia with a T-score of −2.3 in the left femur.

**Conclusions:**

We describe an unusual presentation of SSCD without Tullio phenomenon or Hennebert sign in a population of elderly, East Asian women. There may be an association of SSCD and osteoporosis in this population. Further research is needed to determine the incidence and prevalence of this disorder, as well as the relationship of age, race, osteoporosis risk, and the development of SSCD.

## Background

**Figure 1:**
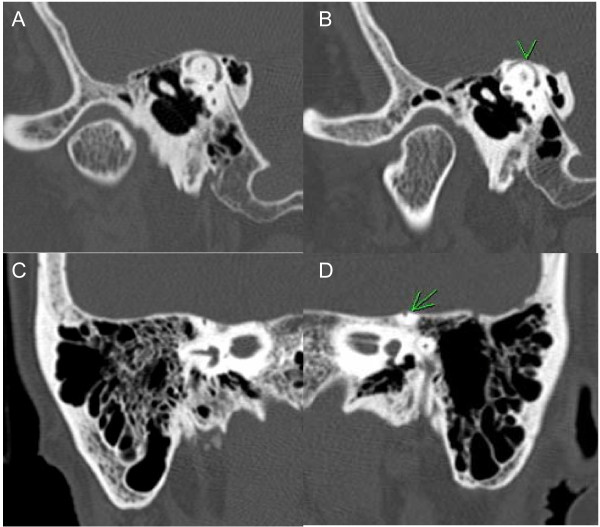
**High-resolution multi-detector CT of the temporal bone was performed with 0.625 mm slices and reformations of the SSC along the longitudinal axis (Pöschl view) and transverse axis (Stenvers view).** To measure the extent of dehiscence, a straight line was drawn subtending the arc of the SSC and rounding to the nearest 0.5 mm in the Pöschl view [3]. In patient 1, the petrous bone overlying the right SSC was eroded, but without dehiscence, as seen on the **(A)** Pöschl view and **(C)** Stenvers view. However, there was dehiscence of the left SSC as seen on the **(B)** Pöschl view (arrowhead) and **(D)** Stenvers views (arrow).

Superior semicircular canal dehiscence (SSCD) is an uncommon cause of vertigo and disequilibrium. The initial population described by Minor *et al.*[1] in 1998 was young, with a median age of 41, and they experienced vertigo or oscillopsia inducible by sound (Tullio phenomenon) and/or increased middle ear or intracranial pressure (Hennebert sign). Computed tomography (CT) of the temporal bone revealed dehiscence of bone overlying one or both of the SSCs [1,2]. The dehisced bone consequently acts as a third window in the inner ear that can disturb the normal pressure gradient directing the endolymphatic flow within the vestibular system [1,3]. Surgical repair by plugging the bony defect can result in improvement of vertigo and disequilibrium in some patients [1,2] Figure 1.

We identified five patients with SSCD from a community health center that serves the East Asian population in Boston. Unlike previous reports, our patients were all elderly women of East Asian descent, had concomitant osteoporosis or osteopenia, and lacked Tullio phenomenon or Hennebert sign.

## Methods

Patients with neurologic symptoms, including those with dizziness, vertigo, and disequilibrium, were evaluated by the neurologist E.T.W. Medical records were reviewed according to a protocol approved by an institutional review board at the clinic. Patients presenting with vertigo, dizziness, or disequilibrium with unclear etiology, as well as those suspected of having unusual inner ear pathology, underwent high-resolution multi-detector CT of the temporal bone, with 0.625 mm slices and reformations of the SSC along the longitudinal axis (Pöschl view) and transverse axis (Stenvers view). To measure the extent of dehiscence, a straight line was drawn subtending the arc of the SSC and rounding to the nearest 0.5 mm in the Pöschl view [3]. If indicated, head CT or magnetic resonance imaging was done to rule out intracerebral or cerebrovascular pathology. T-scores were extracted from bone mineral density (BMD) scans when available.

## Results

Patient characteristics were summarized in Table 1 and 2. Among the 496 individuals seen in the neurology clinic between 2008 and 2010, the median age was 58 (range 20–93) years. SSCD was found in five (1.0%) elderly Chinese women with a median age of 72 (range 57 to 85) years. The prevalence in our cohort is doubled when compared to a large autopsy series of the general population (0.5%) reported to date [4]. Among patients with symptomatic dizziness, vertigo, and disequilibrium (n = 76), the prevalence of SSCD was substantially higher, or 6.6%. Thirty-three of the 76 patients (43.4%) had BMD scans and 27 (35.5%) had documented osteoporosis or osteopenia.

**Table 1:** Characteristics of patients with SSCD and osteoporosis/osteopenia^*^

**Patient**	**Age**	**Gender**	**Inner ear**	**Size of dehiscence**	**Presenting symptoms**	**Hearing loss**	**Tinnitus**	**Dix-hallpike**	**Lowest t-score**^**a**^	**BMD status**
1	85	Female	Left	2.0 mm	New onset dizziness with head movement	None	No	Positive-Left	−2.3 F	Osteopenia
2	70	Female	Left	1.0 mm	Dizziness and falls	None	No	Negative	N/A^b^	Osteoporosis
3	57	Female	Left	1.5 mm	10 year history of vertigo with head movement	None	Yes	Negative	−3.4 S	Osteoporosis
4	81	Female	Left	1.5 mm	Dizziness and falls	Bilateral with AD > AS^c^	No	Negative	−2.9 S	Osteoporosis
5	72	Female	Left	2.0 mm	Dizziness for one year	None	No	Positive-Left	−3.9 S	Osteoporosis

*None of the patients had sound-induced (Tullio phenomenon) or valsalva-induced (Hennebert sign) vertigo.

^a^Lowest BMD T-score, taken from F = femur or S = Spine.

^b^BMD results showed osteoporosis, but T-scores were not available.

^c^Bilateral conductive hearing loss (AD > AS) was detected by Rinnie tuning fork testing at 256 Hz.

**Table 2:** Summary of patient characteristics

**Total number of patients with neurologic symptoms seen between 2008-2010**		**N = 496**
Percent of cohort with dizziness, imbalance or disequilibrium		15.3% (N = 76)
Percent of cohort with dizziness, imbalance or disequilibrium AND with SSCD		6.6% (N = 5)
	Patients with dizziness, imbalance or disequilibrium	Patients with SSCD
N	76	5
Median Age	58 (20–93)	72 (70–85)
Percent female	71.8%	100%
Osteoporosis or Osteopenia (BMD or clinical documentation)	27 (35.5%)	5 (100.0%)

All five individuals had radiological evidence of SSCD confirmed by high-resolution CT of the temporal bone with Pöschl and Stenvers views (Figure 1). None had a history of head trauma, otologic surgery, or active intracerebral disease. Two had histories of intermittent motion-induced vertigo while another two fell secondary to disequilibrium. The vertigo and disequilibrium symptoms were of new onset in three, occurring within 3 months prior to neurological evaluation, while one had a 10-year history of chronic intermittent vertigo. One patient had chronic bilateral hearing loss, while another had tinnitus and vertigo-associated nausea and vomiting. None experienced Tullio phenomenon or Hennebert sign. During neurological examination, two patients had rotatory nystagmus on left-sided Dix-Hallpike maneuver. Although they may have concurrent benign paroxysmal positional vertigo, neither patient derived benefit from Brandt-Daroff exercise or Epley maneuver. The other three patients had normal oculomotor, vestibular, and cerebellar examinations.

All five women were notable for abnormal T-scores on BMD scans. T-scores within three years of SSCD diagnosis were available for four of the five patients; the only patient without T-score values had documentation of osteoporosis in clinical notes prior to receiving care in the clinic. Three of them had definite osteoporosis, with T-scores <−2.5 in the axial spine, while another had osteopenia with a T-score of −2.3 in the left femur (Table 1). Two patients had previously used alendronate for the prevention of fractures from osteoporosis, all for periods of less than 5 years. At the time of SSCD diagnosis, two patients were taking calcium plus vitamin D supplement, while another two used multivitamins that included vitamin D. One patient was taking both ranitidine and omeprazole.

## Discussion

There are major differences between our five elderly, East Asian women with SSCD when compared to cases described in the literature with respect to the demographics and clinical presentations. Our patients had a median age of 75 (range 57 to 85) years, which is significantly older than the median age of 41 (range 13 to 70) years reported in Minor’s review of 65 patients [1]. All of our patients are women while other reports described either a male predominance or equal gender distribution [5,6]. Most importantly, none of our patients experienced Tullio phenomenon or Hennebert sign, while prior report described a high prevalence of these neuro-otologic symptoms, 88% and 63%, respectively [1]. This may be due to the relatively small size dehiscence in our cohort, which were all < 2.0 mm. Yuen *et al.*[3] reported that patients with dehiscence of ≥ 3.0 mm experienced an average air-bone gap hearing loss of 10 decibel on pure-tone audiometry between 500–2000 hertz while none experienced such hearing loss when the dehiscence was < 3.0 mm. Pfammatter *et al.*[6] found that large dehiscence of 2.5 mm or greater were associated with significantly more vestibulocochlear symptoms, including Tullio phenomenon and Hennebert sign. Although not performed, additional testing in our cohort, such as the enlarged, low threshold click-evoked vestibulo-ocular reflex that aligns with the SSC [7] and the large amplitude, low threshold ocular vestibular evoked cervical myogenic potential [8,9], may provide confirmatory physiological evidence of SSCD.

The pathophysiological mechanism giving rise to SSCD in our cohort may be different from the previously reported population, in whom an earlier development of SSCD may be a result of congenital maldevelopment of the petrous bone [1,4]. Notably, all of our patients have osteoporosis or osteopenia and no prior report to date has described abnormal bone mineral metabolism in patients with SSCD. Because Asians are particularly at risk of developing osteoporosis [10], our older patients could have had normal formation of the petrous bone at birth but developed SSCD later in life from the prolonged osteoporotic erosion of bone overlying the SSC. Our view is consistent with the rising prevalence of SSCD or canal thinning in the elderly population, particularly for those at age 80 or older [11]. Furthermore, the slow evolution of dehiscence from osteoporosis may explain the smaller size of the bony defect, ranging from 0.5 to 2.0 mm, in our cohort at diagnosis. The protracted development of dehiscence may also allow time for compensatory adaptation by the nervous system resulting in a paucity of Tullio phenomenon, Hennebert sign, and other severe vestibulocochlear symptoms. However, we cannot exclude the possibility that SSCD and osteoporosis are separate disease processes that co-developed in our cohort.

The incidence and prevalence of SSCD is unknown in a population of patients with vertigo, dizziness, and/or disequilibrium. Autopsy series of cadaver temporal bone revealed an incidence of 0.3% in the general population without neuro-otologic deficits [12]. But among those with inner ear symptoms, the incidence may be as high as 19% in a retrospective series [12]. We found five with SSCD among 76 patients with vertigo, dizziness, and/or disequilibrium, or a prevalence of 6.6%. We suspect that this prevalence is higher than the general population because the clinic, which primarily serves the Asian population in Boston, may allow enrichment of the population at risk for the development of SSCD. Furthermore, improved CT technology may have helped the earlier detection of SSCD. Compared to the 50% sensitivity when 1.0 mm-collimated CT with transverse and coronal images, SSCD was better visualized with 93% sensitivity when 0.5 mm-collimated CT was used together with reformation along the long axis of the SSC [2]. However, further research would be needed to determine the exact incidence and prevalence of SSCD among Asian patients and the general population in the United States, as well as the pathophysiological mechanisms of SSCD in these two groups.

## Conclusions

We documented a case series of elderly, East Asian women with atypical SSCD, as confirmed by high-resolution multi-detector CT of the temporal bone, and concurrent osteoporosis with abnormal T-scores as demonstrated by BMD scans. There may be an association between SSCD and osteoporosis in this susceptible patient population.

## Abbreviations

BMD: Bone mineral density; CT: Computed tomography; SSC: Superior semicircular canal; SSCD: Superior semicircular canal dehiscence.

## Competing interests

The authors declare that they have no competing interests.

## Authors’ contribution

AY drafted the manuscript and worked on data acquisition and analysis; DT drafted the manuscript and analyzed the data; GM analyzed the data; ETW drafted the manuscript and worked on data acquisition and analysis. All authors read and approved the final manuscript.

## Pre-publication history

The pre-publication history for this paper can be accessed here:

http://www.biomedcentral.com/1472-6815/12/8/prepub
